# Live cell imaging of low- and non-repetitive chromosome loci using CRISPR-Cas9

**DOI:** 10.1038/ncomms14725

**Published:** 2017-03-14

**Authors:** Peiwu Qin, Mahmut Parlak, Cem Kuscu, Jigar Bandaria, Mustafa Mir, Karol Szlachta, Ritambhara Singh, Xavier Darzacq, Ahmet Yildiz, Mazhar Adli

**Affiliations:** 1 Department of Physics, University of California at Berkeley, Berkeley, California 94720, USA; 2 Department of Biochemistry and Molecular Genetics, School of Medicine, University of Virginia, Charlottesville, Virginia 22908, USA; 3 Department of Molecular and Cell Biology, University of California at Berkeley, Berkeley, California 94720, USA; 4 Department of Computer Science, University of Virginia, Charlottesville, Virginia 22904, USA

## Abstract

Imaging chromatin dynamics is crucial to understand genome organization and its role in transcriptional regulation. Recently, the RNA-guidable feature of CRISPR-Cas9 has been utilized for imaging of chromatin within live cells. However, these methods are mostly applicable to highly repetitive regions, whereas imaging regions with low or no repeats remains as a challenge. To address this challenge, we design single-guide RNAs (sgRNAs) integrated with up to 16 MS2 binding motifs to enable robust fluorescent signal amplification. These engineered sgRNAs enable multicolour labelling of low-repeat-containing regions using a single sgRNA and of non-repetitive regions with as few as four unique sgRNAs. We achieve tracking of native chromatin loci throughout the cell cycle and determine differential positioning of transcriptionally active and inactive regions in the nucleus. These results demonstrate the feasibility of our approach to monitor the position and dynamics of both repetitive and non-repetitive genomic regions in live cells.

## Abstract (web summary)

CRISPR-Cas9 has been used to image repetitive loci in live cells to explore genome organization. Here the authors demonstrate the imaging of low-repeat regions with a single-guide RNA and the imaging of a non-repetitive region, allowing the tracking of transcriptionally active and inactive regions.

The spatiotemporal organization of chromatin structure plays a critical role in regulating lineage-specific gene expression during cellular differentiation and embryonic development^1^. Global three-dimensional (3D) genome organization and relative gene positioning have been studied primarily using genome-wide technologies, such as chromosome conformation capturing assays^1^. These methods have proven instrumental in identifying long-range intra-genomic interactions and cell type-specific global chromatin states^1^. In addition, fluorescent *in situ* hybridization (FISH)^2^,^3^,^4^ has been used to determine the precise nuclear positions of specific genetic loci in fixed cells. To study chromatin dynamics in living cells, zinc fingers (ZNF)^5^ and transcription activator–like effector (TALE) proteins^6^ have been engineered to target repetitive genomic regions, such as centromeres and telomeres, and track the spatiotemporal dynamics of these regions in live cells. Despite these advances, these approaches are difficult to implement for imaging non-repetitive genomic loci, since they require constructing a large array of TALEs/ZNFs proteins targeting the same locus.

Clustered regularly interspaced palindromic repeats (CRISPR) and CRISPR-associated proteins (Cas) serve as a simpler and more versatile tool for targeting specific DNA sequences in the genome^7^,^8^. In the type II CRISPR system, a Cas9 endonuclease enzyme is targeted to a specific genomic region containing an NGG motif (referred to as the protospacer adjacent motif, PAM) via a 20-nucleotide complementary sgRNA sequence^9^,^10^. The RNA-guidable nature of CRISPR-Cas9 provides greater flexibility over TALEs/ZNFs targeting, and has been repurposed to perform locus-specific genome editing^9^,^10^,^11^,^12^,^13^,^14^,^15^,^16^,^17^ and whole-genome knockout screens^18^,^19^,^20^. Moreover, the catalytically inactive mutant of Cas9 (dCas9) has been used for a wide-range applications including control of gene regulation^21^,^22^,^23^,^24^, purification of specific genomic regions^25^ and whole-genome knockdown screens^26^.

Previous studies have used fluorescently labelled dCas9 for targeting repetitive regions of the genome in live cells^7^. This method has been extended for multiplexed targeting of telomeres and centromeres by co-expression of several dCas9 orthologs fused to different colours of fluorescent proteins and their cognate sgRNAs^27^,^28^. Recently, dual-colour imaging of telomeres and centromeres using modified sgRNAs has also been demonstrated^28^,^29^,^30^,^31^. Targeting dCas9 to a non-repetitive genomic locus is more challenging, because fluorescence signal of a few dCas9-sgRNA complexes at the target region is not sufficient for detection. This bottleneck can be overcome by transfection of cells with 26 unique sgRNAs targeting the same region^7^,^8^. However, this approach is difficult to implement for biological applications due to the challenges in the delivery of dozens sgRNAs into cells and increase in off-target sites by the large number of sgRNAs.

Here we developed an efficient and robust CRISPR-Cas9-based approach to simultaneously image multiple genomic loci in live cells. This method relies on re-engineering an sgRNA containing up to 16 MS2 motifs that bind to the bacteriophage MS2 coat protein (MCP)^32^ and labelling these motifs with fluorescently tagged MCPs. This approach enables targeting of multiple genomic regions in human cells using a single dCas9 and a modular set of sgRNAs. Increasing the copy number of the MS2 sites on a single sgRNA significantly improved the signal-to-noise ratio and reduced the number of dCas9-sgRNA complexes required for reliable detection. Using a single sgRNA, we successfully imaged multiple genomic loci with as few as eight tandem repeats. Non-repetitive genomic regions were detected using as few as 4 separate sgRNAs when imaged using lattice light sheet microscopy (LLSM). Extended sgRNAs also enabled us to track the targeted loci over the course of the entire cell cycle, and to distinguish the nuclear positions of various transcriptionally active and inactive sites in the genome of live cells.

## Results

### Targeting low-repeat-containing loci using a single sgRNA

**Figure 1:**
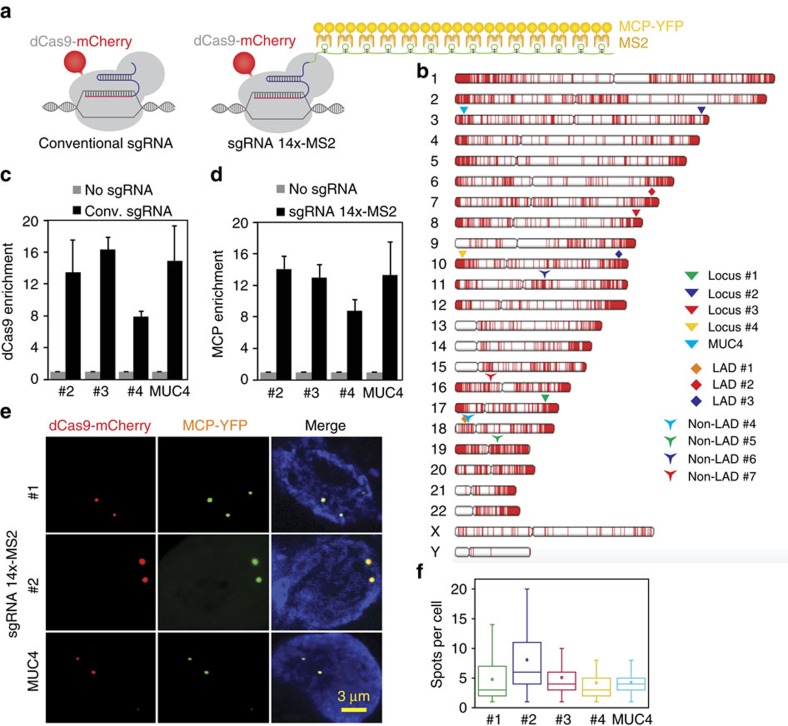
CRISPR-mediated chromatin imaging with conventional and extended sgRNAs. (**a**) Schematics show the conventional sgRNA and an sgRNA extended with 14 MS2 sites. dCas9 is fused to mCherry and MS2 binding sites are decorated with MCP–YFP. (**b**) sgRNA targetable tandem repeats containing four or more unique sites with a PAM sequence (red ticks) for each chromosome of human genome. The genomic loci targeted in this work are shown with larger symbols. (**c**,**d**) ChIP–qPCR results show relative enrichment levels of Flag-tagged dCas9 (**c**) and HA-Tagged MCP–YFP (**d**) at the targeted loci when guided by conventional sgRNA (**c**) and extended sgRNA 14 × -MS2 (**d**). *N*=3 independent experiments. Error bars show s.e.m. (**e**) Representative images show colocalization of MCP and dCas9 spots in the nucleus of HeLa cells transfected with dCas9-mCherry, MCP–YFP and a single sgRNA 14 × -MS2 targeting *MUC4*, locus #1 or #2. Scale bar, 3 μm. (**f**) The number of bright dCas9-mCherry spots detected per cell for each targeted locus. The line and the dot within the boxplot represents the median and the mean, respectively. The outer edges of the box are the 25th and 75th percentiles. The whiskers extend to the minimum and maximum values. *N*_cells_ from left to right are 79, 106, 104, 30 and 57.

We extended Cas9 cognate sgRNAs by inserting up to 14 tandem MS2 stem-loop sequences (Fig. 1a). To test whether the extended sgRNAs (sgRNA 14 × -MS2) form a functional complex with dCas9 while binding to MCP–YFP, we co-transfected HeLa cells with dCas9-mCherry, MCP–YFP, and a single sgRNA 14 × -MS2 targeting an 84-repeat sequence located within the *MUC4* gene (Fig. 1b). Chromatin immunoprecipitation and quantitative PCR (ChIP–qPCR) assays verified that both dCas9 and MCP were substantially enriched at the *MUC4* site (Fig. 1c,d). Notably, the enrichment levels were comparable to that of conventional sgRNAs, indicating that the MS2 sites introduced at the 3′ terminal end of the sgRNA did not interfere with assembly of the dCas9-sgRNA complex at the target region^29^. Confocal imaging of transfected cells revealed colocalization of bright nuclear mCherry and YFP spots (Fig. 1e), indicating that Cas9 and sgRNA localize to the same genomic region.

Next, using a single sgRNA 14 × -MS2, we attempted to target genomic regions containing few repeats^33^. We computationally scanned the genome for 20-nucleotide sequences that can be targeted with an sgRNA. Sequences that repeat multiple times within a 1 kb vicinity of each other and appear only in a single region of the whole genome were analysed further. Notably, we identified >62,000 unique hotspots that contain 4 or more sgRNA-targetable repeats (see Supplementary Data 1 for the complete list of hotspots) with eight repeats on average per hotspot (Supplementary Fig. 1). Next, we selected four unique loci that contained 8, 15, 21 and 33 repeat sequences (locus #1, 2, 3 and 4, respectively, Fig. 1b) and separately transfected HeLa cells with a single sgRNA complementary to each loci (Supplementary Fig. 2). CHIP–qPCR assays confirmed the enrichment of dCas9-sgRNA to these target sites (Fig. 1c,d). In cells that show bright YFP and mCherry spots in the nucleus, every YFP spot colocalized with an mCherry spot (Fig. 1e). Consistent with a previous report^7^, we detected 3.4±0.3 (mean±s.e.m., *N*_cells_=57) spots per cell corresponding to the *MUC4* loci. While a similar number of spots were observed for loci #1, #3 and #4, a higher number of spots were detected using the sgRNA targeting locus #2 (8.0±0.6 mean±s.e.m., *N*_cells_=106, *P*<0.001 for a two-tailed *t*-test, Fig. 1f). Therefore, the extended sgRNA design, in principle, enables targeting of thousands of hotspots in human genome using a single sgRNA.

### Multiplex targeting of low-repeat-containing loci

**Figure 2:**
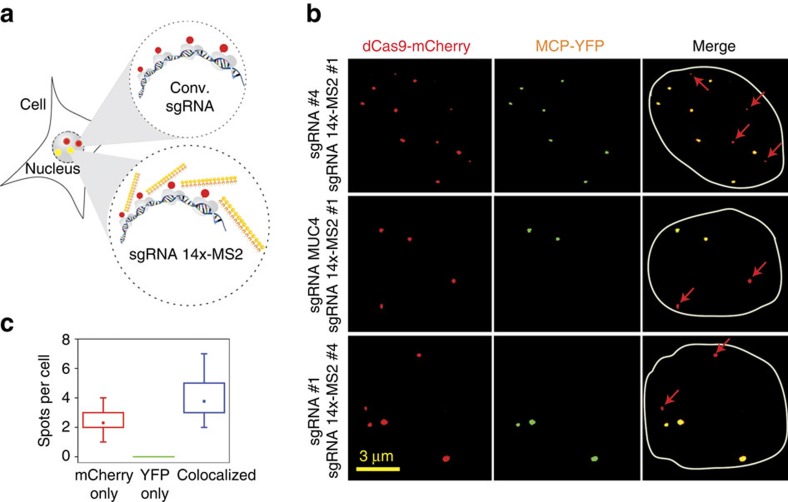
Multicolour imaging of low-repeat-containing loci using a single CRISPR-Cas9 system. (**a**) Cells that are co-transfected with conventional sgRNA and sgRNA 14 × -MS2 targeting separate genomic loci are expected to recruit dCas9-mCherry to both loci while MCP–YFP localizes exclusively to the locus targeted by the extended sgRNA. (**b**) Representative images of HeLa cells co-transfected with dCas9-mCherry, MCP–YFP and conventional and extended sgRNAs targeting separate loci. The merged images show colocalization of YFP and mCherry spots (yellow) along with non-overlapping mCherry spots (red arrows) under three different combinatorial targeting conditions. The nuclear periphery is shown in white in merged images. (**c**) The number of colocalized and non-colocalized mCherry and YFP spots per cell. The dot within the boxplot represents the mean. The outer edges of the box are the 25th and 75th percentiles. The whiskers extend to the minimum and maximum values. In total, 27 mCherry only and 49 colocalized mCherry-YFP spots were observed in 13 cells.

Two separate genomic loci can be differentially labelled by targeting one site using a conventional sgRNA that lacks the MS2 binding site and another site using an sgRNA 14 × -MS2^28^ (Fig. 2a). As a result, the dCas9 signal is expected to appear at both loci, whereas MCP localizes exclusively to the locus targeted by sgRNA 14 × -MS2. While this strategy has been used for imaging highly repetitive telomeric and centromeric sequences simultaneously^28^, dual-colour imaging of low-repeat-containing loci has not been demonstrated.

We transfected HeLa cells with plasmids of dCas9-mCherry, MCP–YFP as well as conventional sgRNA and sgRNA 14 × -MS2 targeting two low-repeat loci. As expected, in cells that show bright YFP and mCherry spots, every YFP spot colocalized with an mCherry spot (Fig. 2b,c), but only 62% of the mCherry spots colocalized with YFP spots. We obtained similar results for each combinatorial targeting of conventional and extended sgRNAs, demonstrating that our findings are not specific to the pair of sgRNAs used (Fig. 2b). These results demonstrate that multicolour labelling of low-repeat-containing genomic loci is feasible with dCas9 using a single extended sgRNA, thus eliminating the need for pooling multiple sgRNAs to achieve sufficient fluorescent signal.

**Figure 3:**
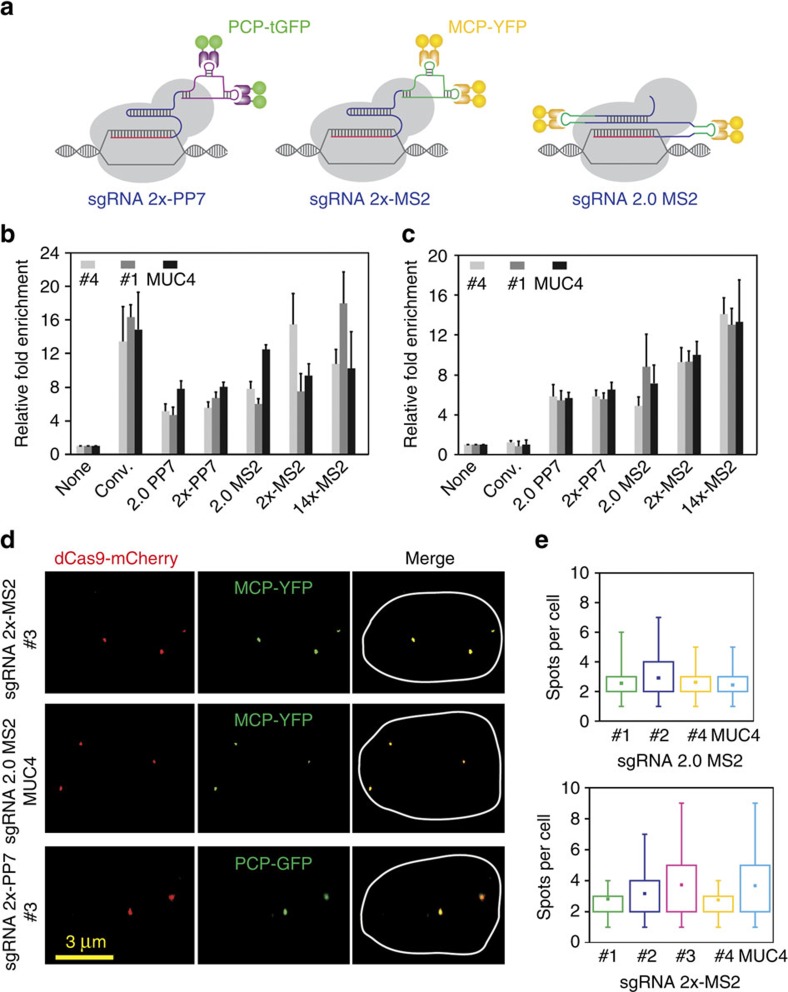
Combinatorial set of CRISPR-derived chromatin-imaging tools. (**a**) Schematics show engineered sgRNAs with various MS2 and PP7 motifs. (**b**,**c**) ChIP–qPCR results show enrichment of Flag-tagged dCas9 (**b**), and HA-tagged MCP– or PCP–YFP (**c**) at the targeted loci when guided by the conventional and engineered sgRNAs. *N*=3 independent experiments. Error bars represent s.e.m. (**d**) Representative images show colocalization of dCas9 and MCP-PCP spots in nucleus of Hela cells transfected with a single engineered sgRNA, dCas9-mCherry and MCP- or PCP–YFP. The nuclear periphery is shown in white in merged images. (**e**) The number of nuclear spots in cells expressing sgRNA 2.0-MS2 (top) and sgRNA 2 × -MS2 (bottom). The dot within the boxplot represents the mean. The outer edges of the box are the 25th and 75th percentiles. The whiskers extend to the minimum and maximum values. *N*_cells_ from left to right are 9, 8, 8, 18 for the top panel and 11, 35, 26, 34 and 53 for the bottom panel.

To further improve the versatility of our dCas9-mediated chromatin imaging platform, we test a variety of different sgRNA designs (Fig. 3a). First, we utilized the high-affinity interaction (similar to the MS2-MCP pair) between the bacteriophage PP7 coat protein (PCP) and its RNA-binding site (PP7) by engineering an sgRNA with 2 × -PP7 sites^28^. Second, by inserting MS2 binding sites into regions of the sgRNA backbone that do not interact with Cas9, we designed sgRNA 2.0 MS2 (ref. 29) to increase the efficiency of dCas9-mediated transactivation complexes^34^. The ChIP–qPCR analysis revealed substantial recruitment of dCas9 and MCP-PCP to the target sites with comparable enrichment levels as conventional sgRNA (Fig. 3b,c).

For each of these sgRNA designs, we co-transfected HeLa cells with plasmids expressing dCas9-mCherry, YFP-tagged MCP-PCP and a single sgRNA targeting a low-repeat-containing locus. In all cases, we observed 100% colocalization of YFP and mCherry spots in the nucleus (Fig. 3d). The number of spots in these cells (Fig. 3e) was significantly less than those that observed using sgRNA 14 × -MS2 (t-test, *P*<0.05, Fig. 1f), suggesting that these sgRNAs are less susceptible to off-target recognition events.

### Targeting of non-repetitive genomic loci

In previous studies, imaging of non-repetitive sites has required the co-expression of as many as 26 unique sgRNAs to achieve sufficient signal-to-noise ratio for spot detection^7^. Because our extended sgRNAs recruit a higher number of fluorescent proteins per dCas9-sgRNA complex, we reasoned that this approach could reduce the number of sgRNAs required to target a non-repetitive locus. This would significantly enhance the efficiency of targeting and imaging native chromatin loci virtually anywhere in the genome of individual cells. To test this possibility, we first attempted to co-transfect HeLa cells with plasmids for dCas9-mCherry and MCP–YFP, along with 30 unique sgRNAs targeting the same locus. However, we were not able to distinguish any cells with bright nuclear spots, due to the low transfection efficiency associated with introducing large numbers of plasmids into a cell.

We aimed to develop a more viable approach for targeting non-repetitive regions in live cells. First, we stably expressed dCas9-mCherry in RPE1, HeLa, U2OS and observed recruitment of dCas9 and MCP to locus #1 in these cell lines (Supplementary Fig. 3). We tested the efficiency of dCas9 imaging to stable dCas9-GFP U2OS cells^7^ when the sgRNA was targeted to the satellite repeat region at centromeres. Plasmid transfection or lentiviral transduction resulted in bright nuclear spots in 60% and 87% of the cells, respectively (*N*_cells_=500, Supplementary Movie 1). However, for the less repetitive *MUC4* locus, lentivirus transduction resulted in discernible nuclear spots within 53% of observed cells (*N*_cells_=260, Supplementary Movie 2), whereas plasmid transfection yielded discernable spots in only 5% of the cells. We also generated a stable U2OS cell line co-expressing both dCas9-GFP and MCP-mCherry (Supplementary Fig. 4a). We targeted the centromeric satellite repeats in this cell-line, and observed both GFP and mCherry spots in 20% of the cells (*N*_cells_=500). All of the GFP and YFP spots colocalized with each other in the nucleus (Supplementary Fig. 4b).

**Figure 4:**
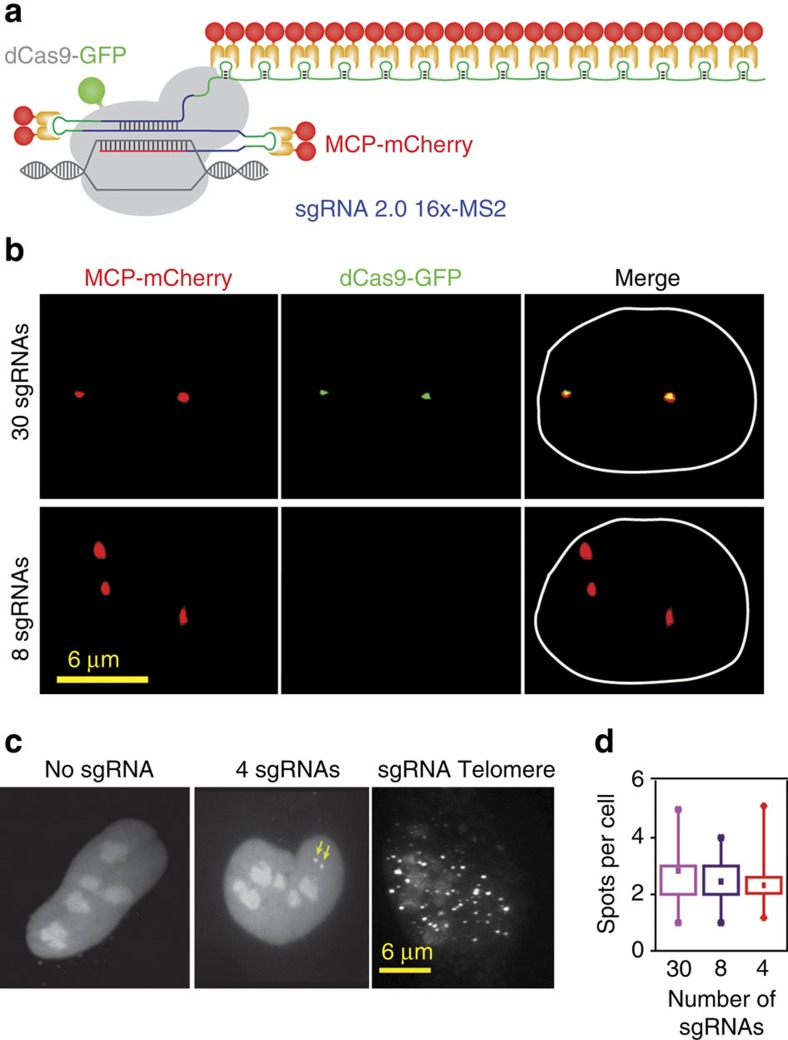
Targeting of a non-repetitive genomic site using four unique sgRNAs. (**a**) sgRNA 2.0 16 × -MS2 was generated by inserting 14x MS2 sites at the 3′ end of sgRNA 2.0. (**b**) Stable U2OS cells expressing dCas9-GFP and MCP-mCherry were transduced with 8 and 30 unique sgRNAs targeting the non-repetitive region in the *MUC4* gene. Confocal imaging of the cells show colocalization of GFP and mCherry with 1–30 sgRNAs, but the dCas9-GFP spots were not detectable with eight sgRNAs. The nuclear periphery is shown in white in merged images. (**c**) Stable U2OS cells expressing dCas9-GFP and MCP-mCherry were transduced with no sgRNA, 4 sgRNAs targeting *MUC4* non-repetitive region and an sgRNA targeting telomeres. mCherry fluorescence was detected using lattice-light sheet microscopy. Bright spots in the nucleus were observed with 1-4 sgRNAs (yellow arrows) and sgRNA telomere, but not in the absence of an sgRNA. (**d**) The number of nuclear spots in cells transduced with 30, 8 and 4 sgRNAs targeting *MUC4* non-repetitive region. The dot within the boxplot represents the mean. The outer edges of the box are the 25th and 75th percentiles. The whiskers extend to the minimum and maximum values. *N*_cells_ from left to right are 12, 11 and 19.

On the basis of these results, we attempted to detect a non-repetitive genomic site in the stable dCas9-GFP-MCP-mCherry U2OS cell line using lentiviral transduction of sgRNA 14 × -MS2s targeting the same locus. We designed 30 different sgRNA 14 × -MS2s that targeted a non-repetitive 5 kb region in the first intron of the *MUC4* gene^7^. Because co-transduction of the cells with these 30 sgRNAs failed to yield any discernable nuclear spots, we re-engineered sgRNA 2.0 with an additional 14 × -MS2 extension at its 3′ end (sgRNA 2.0 16 × -MS2, Fig. 4a). In this case, we were able to detect the colocalization of GFP and mCherry spots in the nucleus using confocal microscopy (*N*_cells_=12, Fig. 4b). However, when the cells were transduced with only 8 of these sgRNA 2.0-16xMS2s, we only observed the mCherry spots, while the GFP spots were no longer detectable (*N*_cells_=11, Fig. 4b). Conversely, the mCherry spots were not detectable in cells transduced with 8 sgRNA2.0 2 × -MS2s, demonstrating that extension of sgRNA 2.0 with additional MS2 binding sites reduces the number of sgRNAs needed for accurate detection of a single genomic locus.

We next attempted to determine the minimum number of sgRNAs necessary to detect a non-repetitive locus. Although cells transduced with eight sgRNAs show detectable nuclear spots in a confocal microscope, we were not able to detect nuclear mCherry spots in cells transduced with four unique sgRNA 2.0 16 × -MS2 targeting *MUC4*. However, when we imaged these cells using LLSM^35^, which has superior light collection sensitivity and lower photobleaching compared to confocal microscopy (Supplementary Fig. 5; Supplementary Movie 3), 19 out of 52 cells showed bright nuclear mCherry spots (2.2±0.3 spots per cell, mean±s.e.m.). As a control, we verified that spots were not visible when cells were mock-transfected in the absence of these sgRNAs (Fig. 4c,d; Supplementary Movie 4). Thus, extension of the sgRNA 2.0 combined with state-of-the-art imaging tools enabled detection of non-repetitive regions in the genome using as few as four unique sgRNAs.

### Long-term imaging of chromatin dynamics

**Figure 5:**
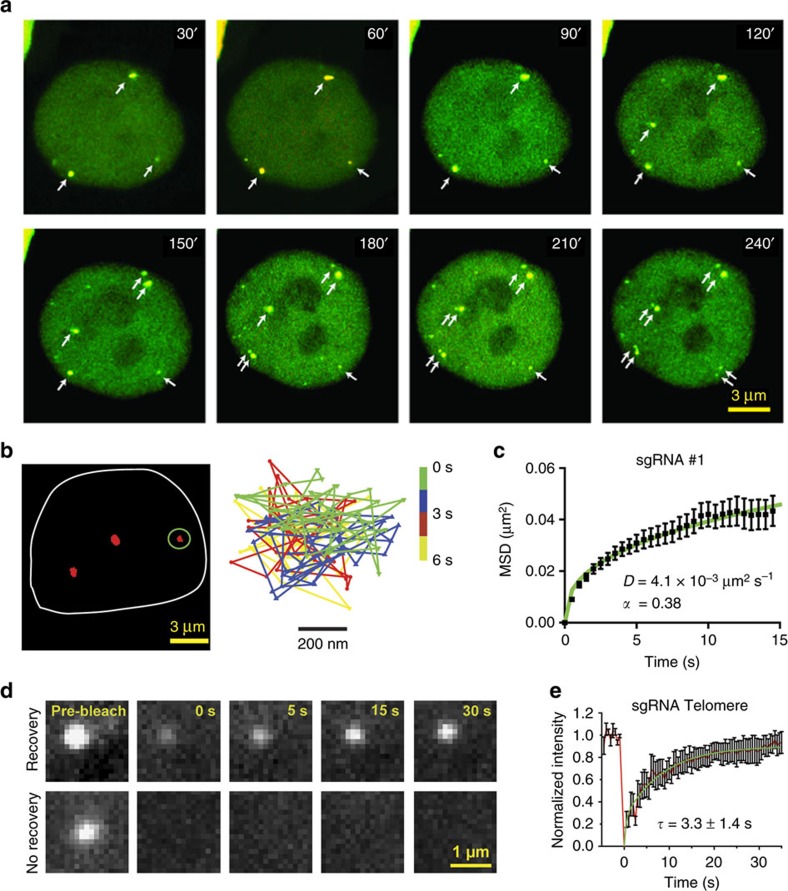
Long-term tracking of specific genomic loci in live cells. (**a**) Snapshots of a 4 h long movie of a HeLa cell co-transfected with dCas9-mCherry, MCP–YFP and sgRNA 14 × -MS2 targeting locus #1. dCas9-sgRNA (yellow spots) remains stably bound to its targeted site throughout the cell cycle. dCas9 spots marked with a single arrow split into two closely spaced loci (double arrows) in different frames. (**b**) A representative image of dCas9-GFP localization to locus #1 in U2OS cells is shown on the left. The nuclear periphery is shown in white. The 2D trajectory of the spot marked with a green ellipse shown on the right. (**c**) MSD plot of dCas9-GFP spots targeting locus #1. The green curve represents a fit to a 2D random walk with a time exponent α (*N*_cells_=42). (**d**) Representative images show partial recovery (top) and no recovery (bottom) of dCas9-GFP spots targeted to telomeres in U2OS cells after photobleaching. (**e**) Fluorescence recovery of dCas9-GFP spots targeting telomeres in U2OS cells. A single exponential fit (green curve) to the fluorescence recovery signal (red curve) reveals the lifetime (*τ*) of the dCas9 recovery (±95% confidence interval, *N*_cells_=30).

The bright fluorescence signal observed at endogenous genomic using the extended sgRNAs enabled us to monitor chromatin dynamics throughout the cell cycle in single cells. First, we performed time lapse imaging of dCas9-GFP targeted to locus #1 in a Hela cell (Supplementary Movie 5). Three bright foci were observed in the nucleus of the cells that were in the G1-S phase. As the cells approached mitosis, each dCas9-GFP spot split into two closely spaced spots (1.2±0.1 μm, mean±s.e.m., *N*=3, Fig. 5a), presumably resulting from the replication of sister chromatids. Interestingly, the three spots were duplicated 30–60 min apart from each other, suggesting that homologous genomic loci are replicated at different time points in different chromosomes (Fig. 5a). Mean squared displacement (MSD) analysis revealed that locus #1 undergoes confined diffusion (*α*=0.38±0.03, ±95% confidence interval, *N*_cells_=42, see Methods) with a diffusion constant (*D*) of 4.1±0.1 × 10^−3^ μm^2^ s^−1^ (±95% confidence interval, Fig. 5b,c; Supplementary Movie 6). Similar results were obtained for dCas9 spots targeting telomeres and *MUC4* (Supplementary Fig. 6a,b)^7^.

We further investigated the binding stability of dCas9-sgRNA to its target locus using fluorescence recovery after photobleaching (FRAP). Recovery of the fluorescence signal was recorded using HiLo microscopy^36^. For dCas9-sgRNA complexes targeting telomeres, 80% of the bleached spots (*N*_cells_=83) did not show any appreciable recovery within a 3-hour time span. The remainder of bleached spots displayed partial recovery with a lifetime of ∼3 s (Fig. 5d,e; Supplementary Movies 7–9). Similar results were obtained for locus #1 and *MUC4* (Supplementary Fig. 6c,d). The spots that exhibited partial recovery may constitute a population of complexes bound to off-target sites, since the mismatches between the sgRNA and the targeted locus are known to reduce the binding stability of Cas9 (ref. 37), resulting in a dynamic exchange between DNA-bound and free dCas9 within the nucleus. Contrary to a recent study that reported a single recovery rate of 12 min for dCas9 targeting telomeres and centromeres^28^, we show that most dCas9-sgRNA complexes stably bind to their genomic targets *in vivo*^38^.

### Nuclear positions of LADs and non-LADs

**Figure 6:**
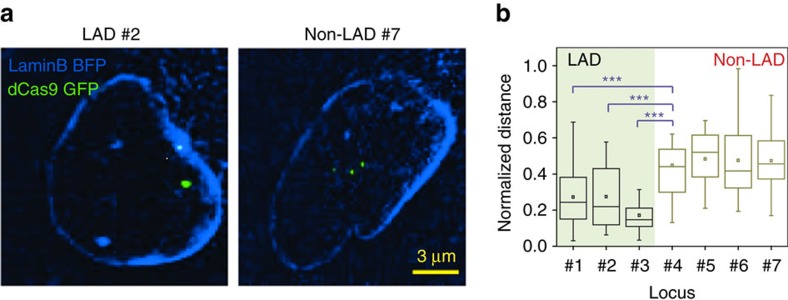
Nuclear positions of transcriptionally active and inactive chromatin loci. (**a**) Representative images of stable dCas9-GFP U2OS cells transduced with an extended sgRNA targeting LAD or non-LAD loci reveal bright spots in nucleus. The nuclear periphery is marked with laminB-BFP. (**b**) The normalized distance of each GFP spot to the nuclear periphery. ***P<0.001 for *t*-test between the mean distance of LAD and non-LAD loci. The line and the dot within the boxplot represent the median and the mean, respectively. The outer edges of the box are the 25th and 75th percentiles. The whiskers extend to the minimum and maximum values. *N*_cells_ from left to right are 69, 38, 19, 23, 21, 17 and 15.

To further test the applicability of our dCas9 imaging platform for visualizing distinct chromatin loci, we studied the spatial organization of transcriptionally active as well as inactive sites associated with nuclear lamina. The LADs are stable heterochromatin genomic regions marked with repressive epigenetic marks^39^. These domains reside at distinct positions within the nucleus^3^,^40^ by forming large-scale ‘topological domains'^41^ that are conserved features amongst many different cell types^1^. Previous studies using FISH experiments have shown that LADs are preferentially located near the nuclear lamina, while non-LADs reside primarily within the interior of the nucleus^39^,^42^. While these fixed-cell experiments have provided considerable insight into the relative intranuclear positioning of these domains discerning the spatiotemporal dynamics of these sites in live cells has remained a considerable technical challenge. To identify suitable targets for our imaging platform, we searched for tandem repeat-containing genomic hotspots that overlapped with previously identified constitutive LAD domains^39^. Next, we selected 3 LADs and 4 non-LAD genomic loci to target with the extended sgRNAs in U2OS cells (Fig. 1b and Supplementary Fig. 7). Chromatin state maps of U20S cells showed that the LAD sequences we target are within a region with high Histone H3 Lysine 9 tri-methylation (H3K9me3) marks, which are associated with stably repressed heterochromatin regions. In contrast, the non-LAD regions are either within or in close proximity to the coding region of a gene marked with high H3 Lysine 36 tri-methylation (H3K36me3), which are associated with active transcriptional elongation (Supplementary Fig. 8). To analyse the relative positioning of the LAD foci, we measured the distance of each fluorescent spot to the nuclear periphery and normalized this distance to the diameter of the nucleus (Fig. 6a; Supplementary Fig. 7). Consistent with a previous report^39^, fluorescent spots from sgRNAs that target LAD domains were located significantly closer (t-test, *P*<0.001) to the nuclear envelope than the non-LAD targeting sgRNAs (Fig. 6b). These results demonstrate the applicability of our approach to study the 3D nuclear organization of specific genomic sites in live cells.

## Discussion

Our imaging platform based on re-engineering the sgRNAs of the CRISPR-Cas9 system from *S. pyogenes* allowed us to image genomic loci with low- or non-repetitive regions with a minimal number of unique targeting sequences. Furthermore, we simultaneously tracked multiple endogenous loci by using the single CRISPR-Cas9 system. By utilizing these flexible sets of chromatin imaging tools separately as well in a combinatorial fashion, we were able to monitor the position and dynamics of distinct chromatin regions. We anticipate that CRISPR-based imaging will simplify future efforts to reveal the dynamic nature of long-range interactions between regulatory genomic regions, such as promoter-enhancer interactions^43^.

Current CRISPR imaging technologies require tandem targeting of multiple sgRNAs to endogenous loci^28^,^44^. However, our approach possesses several unique advantages over recent studies that achieved multicolour chromatin imaging using CRISPR-Cas9 by demonstrating that our method is not mainly limited to imaging highly repetitive regions within the genome. By increasing the copy number of the MS2 sites within a single sgRNA, we substantially improved the fluorescent signal per dCas9-sgRNA complex and detected as few as eight repeat-containing loci using a single sgRNA. Therefore, our methodology potentially enables multicolour imaging of thousands of distinct low-repeat-containing loci within the human genome as well as in other model organisms using a single sgRNA.

Remarkably, the extended sgRNA 2.0 16 × -MS2 design enabled detection of non-repetitive loci with only four unique sgRNAs using LLSM, compared to the 26 sgRNAs reported in previous studies^7^. While it is possible to further reduce the number of sgRNAs required to robustly image a non-repetitive locus, we note that efforts to increase the imaging sensitivity need to be weighed against the potential loss of dCas9 targeting specificity likely to result from significant sgRNA modification. A whole-genome dCas9 mapping studies have revealed a wide range of off-target binding activities exhibited by the CRIPSR-Cas9 system, thus raising an additional technical challenge^45^,^46^. As a result, multiple sgRNAs must be targeted to the same genomic locus in order to form a significantly brighter spot than off-target binding sites and minimize false-positive fluorescent readouts. Future studies will be required to increase the specificity and efficiency of sgRNA targeting by engineering dCas9 variants with reduced off target affinity. Sensitivity in fluorescence imaging of dCas9-sgRNA spots can also be further improved using split fluorescent proteins^47^ and novel protein labelling approaches^48^. Despite these future challenges, we expect that our extended sgRNA-dCas9 imaging method will serve as an important step towards directly visualizing dynamics of single genomic loci at high spatiotemporal resolution.

## Methods

### Plasmid construction

*S. pyogenes* Cas9 gene carrying D10A and H840A mutations (dCas9) was fused to 2x SV40 nuclear localization sequence (NLS) and 3 × -Flag-tag at the N terminus and with mCherry or GFP at the C terminus. dCas9 constructs were cloned into a lentiviral vector containing an inducible promoter pTRE3G (Tet-on 3G inducible expression system, Clontech). MCP and PCP were fused with an HA-tag and the SV40 NLS at the N terminus and with YFP or GFP at the C terminus. For imaging of non-repetitive loci, MCP-mCherry was cloned into the pHR lentivirus backbone (Addgene #46911) and expressed by the SFFV promoter.

sgRNAs with 2 × -MS2, 14 × -MS2, 2.0 MS2, 2.0 16 × -MS2 and 2 × -PP7 extensions were cloned into the pSLQ1661-sgMUC4-E3(F+E) (Addgene #51025) plasmid. sgRNAs 14 × -MS2 and 2.0 16 × -MS2 contain A–U flip and hairpin extension to increase targeting efficiency^7^. mCherry under the control of CMV promoter was removed from the original plasmid backbone and TagBFP was introduced into the backbone as a transduction indicator. sgRNAs were expressed under the U6 promoter. sgRNA sequences were amplified from an oligo template with the same 3′ primer and a unique 5′ primer that contain the 20 nucleotides targeting sequence. See Supplementary Table 1 for the sequences of these primers and oligo templates used. The sgRNA fragments and the plasmid backbone were digested with BstXI-BamH1. The ligation was performed by controlling the molar ratio between plasmid and the sgRNA insert 1:500 at 37 °C for 15 min. The ligation solution was transformed in Stbl3 competent cells.

### Cell culture

Human embryonic kidney (HEK293T), human osteosarcoma (U2OS), retinal pigment epithelium (RPE) and human cervical cancer (HeLa) cells were obtained from University of California Berkeley Tissue Culture Facility. No cell lines used in this study were found in the database of commonly misidentified cell lines that is maintained by ICLAC and NCBI Biosample. The cell lines were not authenticated and not tested for mycoplasma contamination. The cells were maintained in DMEM in 10% FBS (Clontech). All cells were maintained at 37 °C and 5% CO_2_ in a humidified incubator.

### Lentiviral production and transduction

For viral production, HEK293T cells were seeded into T75 flask 1 day before transfection. 6 μg of pMD2.G plasmid, 9 μg of psPAX plasmid and 15 μg of the lentiviral vector that expresses Tet-on 3G, dCas9 or MCP were co-transfected into HEK293T cells using FuGENE-6 (Promega) following the manufacturer's recommended protocol. Media was refreshed 12 h after transfection. Virus was collected 24 and 48 h after first media refreshment, filtered through 0.45 μm filter, flash-frozen with liquid nitrogen and stored at −80 °C. For viral transduction, cells were incubated with virus solution diluted threefold in DMEM medium and supplemented with 10 mg ml^−1^ polybrene for 24 h. The transduction efficiency was verified with BFP fluorescence using Zeiss AxioObserver epi-fluorescent microscope. To make a stable cell line, cells were transduced with lentivirus, selected with appropriate antibiotics (4–6 μg ml^−l^ puromycin or blasticidin) for 3 days and sorted with fluorescence-activated cell sorting (FACS). The medium containing dead cells were replaced every day. See Supplementary Tables 2–4 for the lists of the plasmids, sgRNAs, lentiviruses and stable cell lines used in this study.

### Plasmid transfections

Cells were plated on 35 mm diameter glass bottom MatTek plates. dCas9-GFP was expressed under the Tet-on promoter in the absence of doxycycline treatment. For regular cell lines, the ratio of plasmids containing dCas9-mCherry and sgRNA (0.25 μg dCas9-mCherry, 2.5 μg sgRNA) was adjusted to obtain minimum background and to enhance the dCas9-sgRNA assembly^27^. Cells were imaged 24 h following transfection.

### ChIP assay

HeLa cells were plated on three 10-cm plates and transfected with the plasmids that express dCas9, MCP and sgRNA. Transfected cells were cross-linked with 1% formaldehyde for 10 min and neutralized with 125 mM glycine for 5 min at 37 °C. Pellets were lysed in SDS lysis buffer and incubated for 20 min on ice. The chromatin was sonicated using Branson digital sonifier for 9 min at 40% amplitude with 0.7 s ‘on' and 1.3 s ‘off' pulse cycles. Fragmented chromatin was diluted with ChIP-dilution buffer (16.7 mM Tris-HCl pH 8.1, 0.01% SDS, 1.1% Triton X-100 and 1.2 mM EDTA) and incubated with 1.5 μg HA-Chip grade antibody (Abcam, #9110) overnight at 4 °C. After overnight incubation, 30 μl mixture of protein A-G magnetic beads (Dynabeads, Life Technologies) was added to lysates and rotated for 2 h at 4 °C. Next, beads were washed twice on the magnetic field with low-salt immune complex wash buffer (20 mM Tris-HCl pH 8.1, 0.1% SDS, 1% Triton X-100, 2 mM EDTA, and 150 mM NaCl); LiCl wash buffer (10 mM Tris-HCl pH 8.1, 250 mM LiCl, 1% NP40, 1% deoxycholate and 1 mM EDTA); and TE (10 mM Tris-HCl pH 8.0 and 1 mM EDTA). The chromatin was recovered from the beads by 30 min incubation with elution buffer (100 mM NaHCO_3_ pH 8.0, 0.2% SDS and 5 mM DTT) at 65 °C (refs 49, 50). After reverse cross-linking, proteinase K and RNase digestion, DNA was extracted with ethanol precipitation and quantified via Qubit Fluorimeter. The relative enrichment levels of genomic regions of interest within the ChIP-DNA were assessed by qPCR amplification with specific primers that were designed to amplify ∼100 bp around the sgRNA targeting sites.

### Computational identification of hotspots in human genome

Regions that contain four or more identical sgRNA target sites that are not present anywhere else in the genome were defined as ‘genomic hotspots'. All 20-nucleotide-long sequences ending with a PAM sequence (NGG) at the 3′-end were initially identified from the human genome (hg19). Sequences with GC content lower than 35% or higher than 80% were excluded from the analysis. Among these sgRNA targetable genomic sites, the exact matching sequences across different chromosomes and sequences with less than four repeats in genome were excluded from the analysis. The remaining sequences were sorted and distances between the adjacent sequences were calculated using the BEDTools suite. The sequences that are separated by more than 10 kb were removed from the analysis. The identical 20-nucleotide sgRNA-target sequences that are present within a maximum of 10 kb genomic distance to each other were analysed for the number of repeats.

### Microscopy

Confocal imaging was performed using Zeiss LSM 710 confocal microscopy with a × 63 1.4 numerical aperture (NA) oil immersion objective at 37 °C with 5% CO_2_. 405, 488 and 561 nm excitation beams were used to image DAPI, YFP-GFP and mCherry, respectively. To minimize the bleed through of fluorescent signal from YFP-GFP to mCherry channels, time sharing between 488 and 561 nm excitation and image collection was used. Digital gain and offset adjustment on PMT are optimized to use the full dynamic range of the detector. The *z* sectioning step was set to 0.64 μM and the range of sectioning was adjusted manually. The *z*-stacks were converted to 2D projections in Image J.

For single particle tracking assays, cells were imaged using a custom-built HiLo microscope, equipped with Nikon TiE microscope body, × 100 1.49 NA oil objective, Nikon perfect focusing system, electron-multiplied charge coupled device (EM-CCD camera (Andor) and 405, 488 and 561 nm lasers (Coherent). The power of 488 and 561 beams were 160 and 53 W cm^−2^ for GFP and mCherry excitation, respectively. Sample heater was used to maintain the culture temperature at 37 °C. The frame rate of imaging was set to 10 Hz. The movies were analysed in ImageJ.

A custom-built LLSM based on the previously described instrument design^35^ was used. Briefly, the light sheet was generated from the interference of an array of 30 beams in a square lattice, and dithered to create a uniform excitation sheet. The inner and outer numerical apertures of the excitation sheet were 0.505 and 0.60, respectively. The cells were plated on 5-mm diameter coverslips and media was replaced with PBS before imaging. The coverslips were sonicated three times with 57% ethanol and 10% sodium hydroxide, then washed with distilled water. The coverslips were then sterilized with ultraviolet for 30 min, coated with 1% weight per volume polylysine solution for 2–24 h and washed with cell culture medium before usage. The sample was excited with 500 μW 488 nm beam and 100 μW 590 nm beam for GFP and mCherry imaging, respectively. The laser power was measured at the back aperture of the objective. The sample was scanned in the *z* axis of the imaging objective with a slice interval of 200 nm over a 20 μm range. Movies were acquired at 100 ms per slice using a pair of Andor iXon Ultra 897 EMCCDs for dual-colour imaging. Three-dimensional projection of the acquired movies was generated using maximum intensity in ImageJ.

### FRAP assay

Cells were imaged using the custom-built HiLo microscope equipped with a piezo-scanning mirror (Physik Instrumente) to steer a focused 488 nm laser beam in the image plane. Optical pathways of excitation and photobleaching beam were controlled separately by Uniblitz shutters using custom software written in Labview. Fluorescence spots were detected using 160 W cm^−2^ HiLo excitation and photobleached by 0.7 MW cm^−2^ focused laser beam. Time lapse of 1 min between frames was used to minimize photobleaching of the fluorescent spots during a slow recovery. The integrated intensity of chromosome loci was calculated with the Time Series Analyzer plugin of ImageJ and corrected for the changes in the background fluorescence. The calculated intensity was analysed with MATLAB. The fluorescence intensity of each spot that shows FRAP recovery was normalized before taking their average. The recovery rate was calculated using the easyFRAP software^51^.

### Data analysis

Individual dCas9-GFP or -mCherry spots were imaged using the HiLo microscopy at 30 Hz frame rate and 200 kW cm^−2^ excitation power. The fluorescent spots in live cells were detected using a 2D Gaussian fitting algorithm written in Python. The majority of the spots were diffraction limited with the full-width at half maximum of 252±106 nm (mean±s.d., *N*=1,850). On average, 1,580 photons were detected from each spot per frame. The spots with a full-width at half maximum between 120 and 420 nm and a minimum 800 photons detected per frame were analysed further, and the rest of the spots were excluded from data analysis.

The position of spots was tracked by fitting their intensity profile to a 2D Gaussian equation using the ImageJ plugin, ThunderStorm. The MSD is calculated from the 2D particle tracking data. D was calculated by 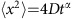, where *α*<1 and =1 indicate confined and free diffusion, respectively. To account for the drift of the microscope stage and cell movement in long-term tracking experiments, only the pairwise distances between the spots have been used in the MSD analysis^52^.

To evaluate the transcriptional state of the LAD and non-LAD domains in U20S cells, we analysed ENCODE chromatin state maps for H3K9me3 (GEO: GSM788078) and H3K36me3 (GEO: GSM788076) marks.

### Data availability

All relevant data are available from the authors upon request. Chromatin state maps used in analyses are available from the GEO with accession numbers GSM788078 and 788076.

## Additional information

**How to cite this article:** Qin, P. *et al*. Live cell imaging of low- and non-repetitive chromosome loci using CRISPR-Cas9. *Nat. Commun.* **8,** 14725 doi: 10.1038/ncomms14725 (2017).

**Publisher's note**: Springer Nature remains neutral with regard to jurisdictional claims in published maps and institutional affiliations.

## Supplementary Material

Supplementary Information Supplementary Figures and Supplementary Tables

Supplementary Movie 1 Transduction efficiency of lentivirus targeting centromere. Stable dCas9-GFP U2OS cells were transduced with an sgRNA lentivirus targeting centromeric satellite repeats. The cells were imaged using HiLo microscopy and moved to different fields of view manually. The scale bar is 9.6 μm.

Supplementary Movie 2 Transduction efficiency of lentivirus targeting MUC4 repetitive region. Stable dCas9-GFP U2OS cells were transduced with an sgRNA lentivirus targeting an 84-repeat sequence in the MUC4 gene. The cells were imaged using HiLo microscopy and moved to different fields of view manually. The scale bar is 9.6 μm.

Supplementary Movie 3 Lattice light sheet imaging of telomeres in a stable dCas9-GFP U2OS cell. U2OS cells stably expressing dCas9-GFP and MCP-mCherry were transduced with an sgRNA lentivirus targeting telomeres. The cells were imaged under lattice light sheet microscopy at 100 ms per frame. The scale bar is 6 μm.

Supplementary Movie 4 Lattice light sheet imaging of MUC4 non-repetitive region with 4 sgRNA 2.0 16x-MS2. U2OS cells stably expressing dCas9-GFP and MCPmCherry were imaged using lattice light sheet microscopy at 100 ms per frame. The left panel shows a control stable cell without sgRNA transduction and the cell shown in the right panel was transduced with four unique sgRNA 2.0 16x-MS2 lentivirus targeting MUC4 non-repetitive region. The dCas9-GFP signal is not observable and only MCPmCherry signal is shown. The scale bar is 6 μm.

Supplementary Movie 5 Long term imaging of dCas9-sgRNA complexes localized to locus #1 in a stable dCas9-GFP U2OS cell. Cells were transduced with sgRNA #1 lentivirus and imaged with HiLo microscopy at 50 ms per frame. The scale bar is 6 μm.

Supplementary Movie 6 Real time observation of replication of genomic loci in different chromosomes in HeLa cells. Cells were co-transfected with sgRNA 14x-MS2 #1. dCas9-mCherry, and MCP-YFP and imaged using scanning confocal microscopy at every 15 minutes. DNA replication of the same genomic locus in different chromosomes was observed in different frames. See Figure 5a for the analysis of this movie. The scale bar is 3 μm.

Supplementary Movie 7 Single particle tracking of dCas9-mCherry localized to locus #1 in a HeLa cell. Cells were co-transfected with an sgRNA 14x-MS2 targeting locus #1, dCas9-mCherry and MCP-YFP, and imaged using scanning confocal microscopy at 100 ms per frame. Tracking of each spot to a 2D Gaussian is shown per frame and center of the Gaussian is highlighted with a colored circle. The scale bar is 6 μm.

Supplementary Movie 8 FRAP measurements of dCas9-GFP localized to telomeres with partial recovery in stable dCas9-GFP U2OS cells. Cells were transduced with sgRNA telomere lentivirus and imaged with HiLo microscopy at 300 ms per frame. Telomeres highlighted with colored ellipses were photobleached using a focused 488 nm beam. Telomeres marked with green ellipses did not show any detectable recovery over the course of the movie. The telomere marked with a red ellipse showed partial recovery. Scale bar is 6 μm.

Supplementary Movie 9 FRAP measurements of dCas9-GFP localized to telomeres without recovery in stable dCas9-GFP U2OS cells. Cells were transduced with sgRNA telomere lentivirus and imaged with HiLo microscopy at 300 ms per frame. Telomeres highlighted with a red ellipse were photobleached using a focused 488 nm beam. No recovery has been observed for these spots. Scale bar is 6 μm.

Supplementary Data 1 The list of hotspots in human genome.
