# Supplementary material for: Live cell imaging of low- and non-repetitive chromosome loci using CRISPR-Cas9
Source: Nat Commun. 2017 Mar 14;8:14725. doi: 10.1038/ncomms14725 (PMC5424063; doi:10.1038/ncomms14725)
Supplement: Supplementary Information — Supplementary Figures and Supplementary Tables [file ncomms14725-s1.pdf]

1. Supplementary Tables

Gene	Selection Marker
spdCas9-mCherry	Puromycin
spdCas9-GFP	Puromycin
MCP-YFP	Blasticidin
MCP-mCherry	Blasticidin
PCP-YFP	Blasticidin

Supplementary Table 1: The list of plasmids used in this study.

Number	Designed sgRNA type	Target site	sgRNA guide sequence
Locus#1	2x-PP7<br>2.0 MS2<br>2x-MS2<br>14x-MS2<br>2.0 16x-MS2	Chr17:82448279-82448298	GACCAGGGAGGAGGAGACAT
Locus #2	2x-PP7<br>2.0 MS2<br>2x-MS2<br>14x-MS2<br>2.0 16x-MS2	Chr3:196463040-196463059	GAGAGTTGAGTCTGTACTGT
Locus #3	2x-PP7<br>2.0 MS2<br>2x-MS2<br>14x-MS2<br>2.0 16x-MS2	Chr8:144767945-144767964	GTAAGGTTCAGACTCTGGCT
Locus#4	2x-PP7<br>2.0 MS2<br>2x-MS2<br>14x-MS2<br>2.0 16x-MS2	Chr10:1047031-1047050	GTGTAGACAGTGGAGCAGCT
Locus MUC4	2x-PP7<br>2.0 MS2<br>2x-MS2<br>14x-MS2<br>2.0 16x-MS2	Chr3:10038-10057	GACCTGTGGATGCTGAGGAA
LAD #1	Conv.	Chr18:6272463-6272482	GAACCACCAGTTTAATGCAG
LAD#2	Conv.	Chr7:154661302-1544661321	GGTGAATCACCATGGCGTAT
LAD #3	Conv.	Chr10:127396940-127396959	GATCTGAGCATGAGTTACAC
Non-LAD#4	14x-MS2	Chr18:9815563-9815582	GATGGAGGACAGCATCTACA
Non-LAD#5	14x-MS2	Chr19:32745723-32745742	GGGAGAGAGACTGGCTGATG
Non-LAD#6	14x-MS2	Chr11:69068864-69068883	GTCACTTCCTAGGACTCAGA
Non-LAD#7	14x-MS2	Chr16:29484745-29484764	GACACCTGCCGAGCGTCTGC
centromere	2.0 MS2<br>14x-MS2<br>2.0 16x-MS2		GAATCTGCAAGTGGATATT

Telomere	2.0 MS2<br>14x-MS2<br>2.0 16x-MS2		TTAGGGTTAGGGTTAGGGTTA
sgMuc4-1	2.0 16x-MS2		GTAAAGTAGAAAAGGCATAAA
sgMuc4-2	2.0 16x-MS2		GAACCCGGAATGGCACTTGTGT
sgMuc4-3	2.0 16x-MS2		GCTCGCCTCGGCTCCCAAAGTGC
sgMuc4-4	2.0 16x-MS2		GAACAGAGGGCCAGAGAGCAGCC
sgMuc4-5	2.0 16x-MS2		GTACACCCTTGTGTACAGAGCT
sgMuc4-6	2.0 16x-MS2		GTTCCTTTTGGCTCCCTGAAG
sgMuc4-7	2.0 16x-MS2		GAAGAGTGGAGGCCGTGCGCGG
sgMuc4-8	2.0 16x-MS2		GCAAGCAAGGGAAGCGACAAGG
sgMuc4-9	2.0 16x-MS2		GTAGCCCCGGCATTGGCCTT
sgMuc4-10	2.0 16x-MS2		GCATATTTGAGGAGCTTCC
sgMuc4-11	2.0 16x-MS2		GGCTGCAAGAGAAGCCATGC
sgMuc4-12	2.0 16x-MS2		GATGTTTCAGGACTAGGCTGA
sgMuc4-13	2.0 16x-MS2		GAGGCTGGGGCTTGGGGCGCC
sgMuc4-14	2.0 16x-MS2		GCCCTGCCCCGTGTCTCCCC
sgMuc4-15	2.0 16x-MS2		GCTGAGAGCTGCATTTCGAA
sgMuc4-16	2.0 16x-MS2		GAATGAATGGCTGTCTCAGCA
sgMuc4-17	2.0 16x-MS2		GTCCAGTGGCCAGTGGATTTTG
sgMuc4-18	2.0 16x-MS2		GTAGAGATGCCGCCCCGCCC
sgMuc4-19	2.0 16x-MS2		GGGCATTTGTGTTGCACGTG
sgMuc4-20	2.0 16x-MS2		GACAGAGTTTCTCTCTGTCCCCC
sgMuc4-21	2.0 16x-MS2		GACTCAATTTCTCAGAACATGCTG
sgMuc4-22	2.0 16x-MS2		GCTAAGGACAAGAGGCAATGAG
sgMuc4-23	2.0 16x-MS2		GGCTTGGTGTATTCAGAATG
sgMuc4-24	2.0 16x-MS2		GCTCCCTGCAACCTCTGCCTCCC
sgMuc4-25	2.0 16x-MS2		GTCCAGCATCAGCGACGCCCT
sgMuc4-26	2.0 16x-MS2		GCCACAGCGCACTCCACGGGGAA
sgMuc4-27	2.0 16x-MS2		GTTTCCTTAAGGAACAGCCC
sgMuc4-28	2.0 16x-MS2		GGAGCTGGGCCAGGAGAGGAGA
sgMuc4-29	2.0 16x-MS2		GAGCGCAGAGGGGCAAGACCT
sgMuc4-30	2.0 16x-MS2		GCTGGACACTCAGCTCCATG

Supplementary Table 2: The list of sgRNA expressing plasmids and their target sequence used in this study.

Cell type	Stable expression
HeLa	dCas9-mCherry
U2OS	dCas9-mCherry
U2OS	dCas9-GFP
U2OS	dCas9-GFP/MCP-mCherry
RPE1	dCas9-mCherry

Supplementary Table 3: Stable cell lines used in this study. All of the cell lines, except the stable dCas9-GFP U2OS cell line, were generated in this study.

Primer	Sequence
pLJM1-EGFP-cloning-forward	5'CCGTCAGATCCGCTAGCGCTACCGGGGGCCACCATGGCGCCAAAAAAG 3'
pLJM1-EGFP-cloning-reverse	5' GCCATTTGTCTCGA GGTCGAGAATTTTACTTGTACAGCTCGTCC 3'
pHR SSFV-forward	5' GTCAGCGGCCGCCTTTACTTGTACAGCTCGTCC3'
pHR SSFV-reverse	5' GTCAGGATCCGCCGGGCCACCATGGCGCCAAAAAAG 3'
hUbc-dCas9-forward	5' CATGATCGATCTTTACTTGTACAG3'
hUbc-dCas9-reverse	5' CATGATCGATCTTTACTTGTACAG 3'
sgRNA backbone	5'GTTTAAGAGCTATGCTGGAAACAGCATAGCAAGTTTAAATAAGGCTAGTCCGTTATCAACTTGAAAAAGTGGCACCGAGTCGGTGCGGATC 3'
sgRNA 2.0 backbone	5'GTTTAAGAGCTATGCTGGCCAACATGAGGATCACCCATGTCTGCAGGGCCCAGCATAGCAAGTTTAAATAAGGCTAGTCCGTTATCAACTTGGCCAACATGAGGATCACCCATGTCTGCAGGGCCAAGTGGCACCGAGTCGGTGC3'
sgRNA forward	5' GGAGAACCACCTTGTTGG N_{17-23} GTTTAAGAGCTATGCTGGAAACAGCA 3'
sgRNA reverse	5' CTCAGGATCCGCACCGACTCGGTGCCACTTTTTC3'
sgRNA2.0 forward	5' GGAGAACCACCTTGTTGGN_{17-23} GTTTAAGAGCTATGCTGGGCCAAC3'
sgRNA2.0 Reverse	5' CCTTAGGATCCGCACCGACTCGGTGCCACTTTTTC 3'

Supplementary Table 4. The list of primers used to generate the sgRNAs.

2. Supplementary Figures

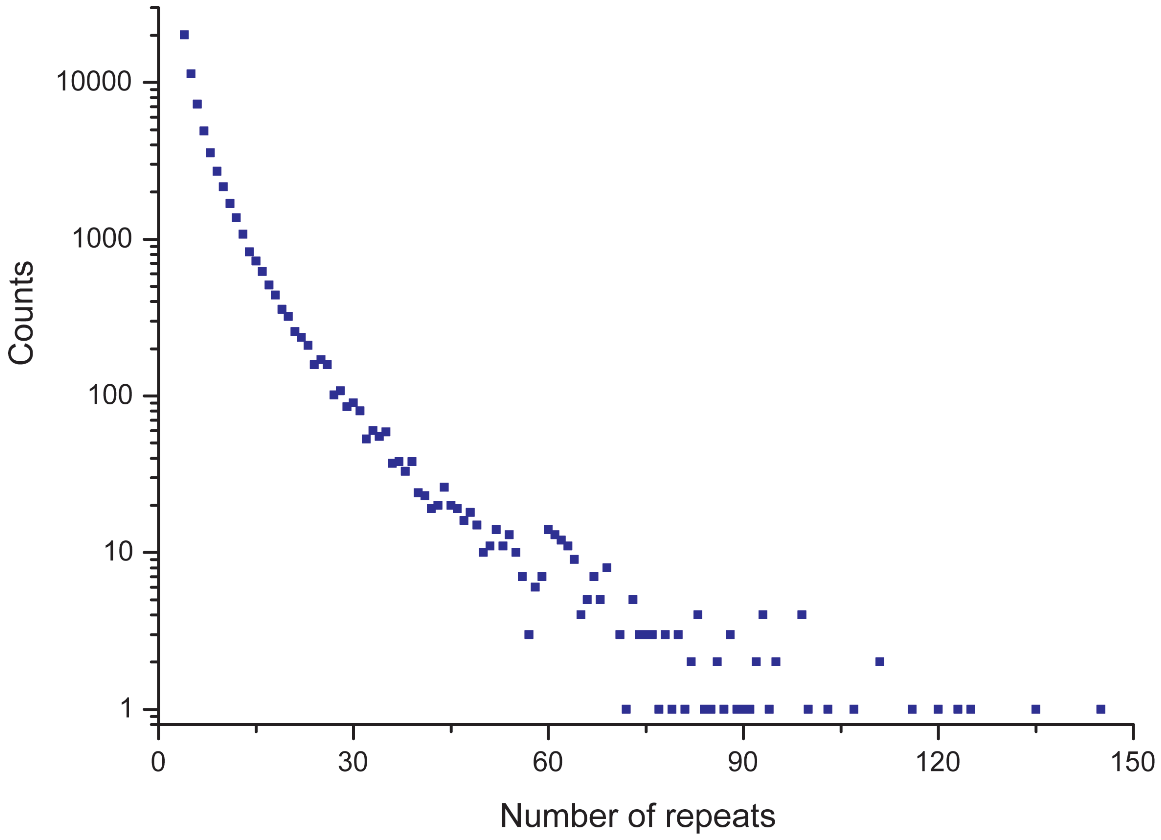

Supplementary Figure 1: The histogram of hotspots in human genome as a function of the number of sgRNA repeats. See Supplementary Data 1 for the complete list of the hotspots.

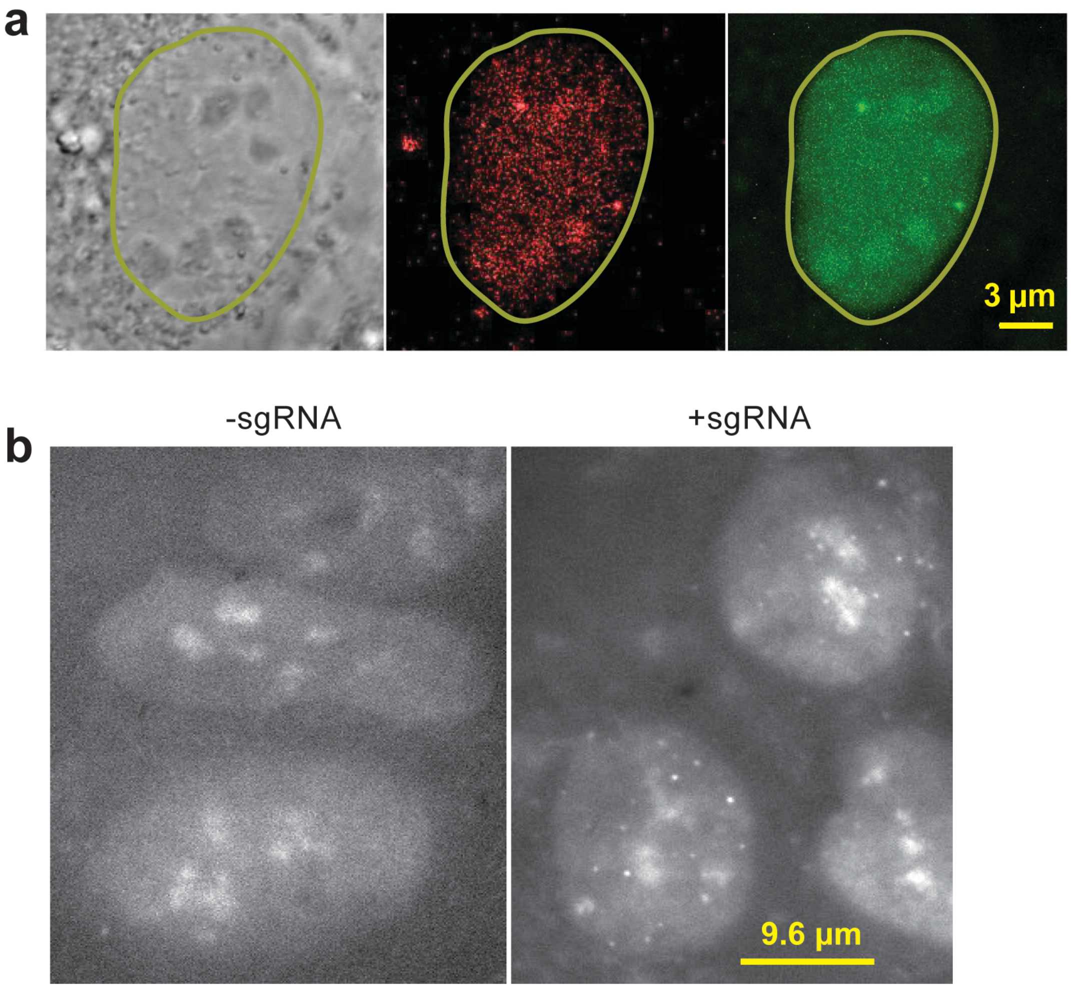

Supplementary Figure 2. Transfection of mammalian cells with an sgRNA and determination of the nuclear periphery. (a) Nuclear periphery of the HeLa cell was determined by brightfield imaging (left), the background of the dCas9-mCherry signal (middle) and the background of the MCP-YFP signal (right). **(b)** Representative images of stable dCas9-GFP U2OS cells show bright nuclear spots when transfected with an sgRNA targeting telomeric repeats. Spots are not observed in the absence of an sgRNA.

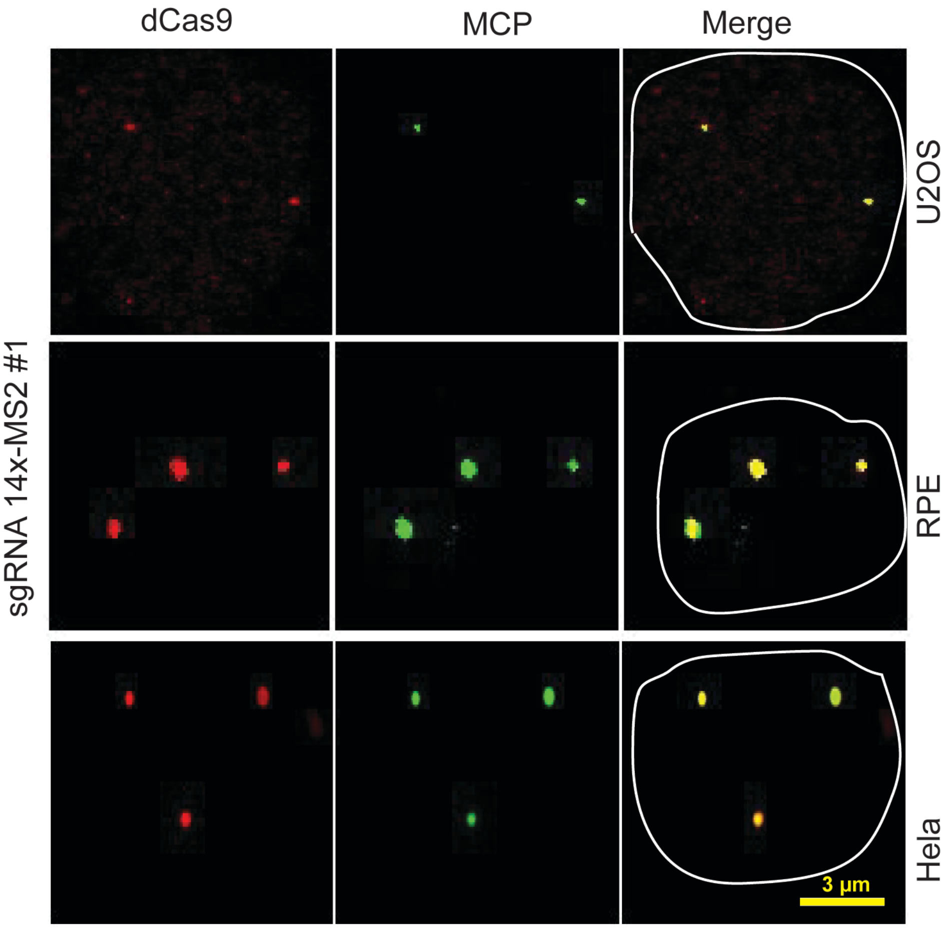

Supplementary Figure 3: Two-color imaging of locus #1 using sgRNA 14x-MS2 in cell lines stably expressing dCas9-mCherry. The cells were co-transfected with MCP-YFP and a single extended sgRNA. Nuclear periphery of the cells was marked with white in merged images.

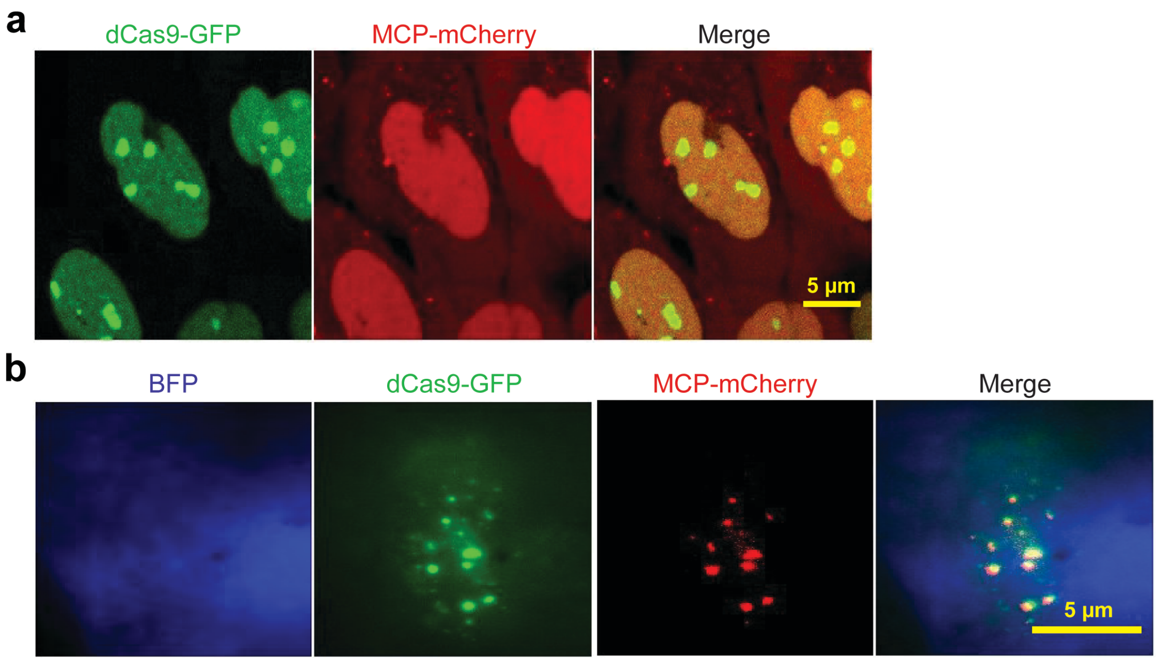

Supplementary Figure 4: Two-color imaging using the stable dCas9-GFP MCP-mCherry U2OS cell line. (a) The cells show both dCas9-GFP and MCP-mCherry signal in the nucleus. **(b)** Transduction of these cells with sgRNA 2.0 16x-MS2 lentivirus targeting centromeric satellite repeats results in colocalization between dCas9 and MCP spots. BFP signal was used as a transduction control.

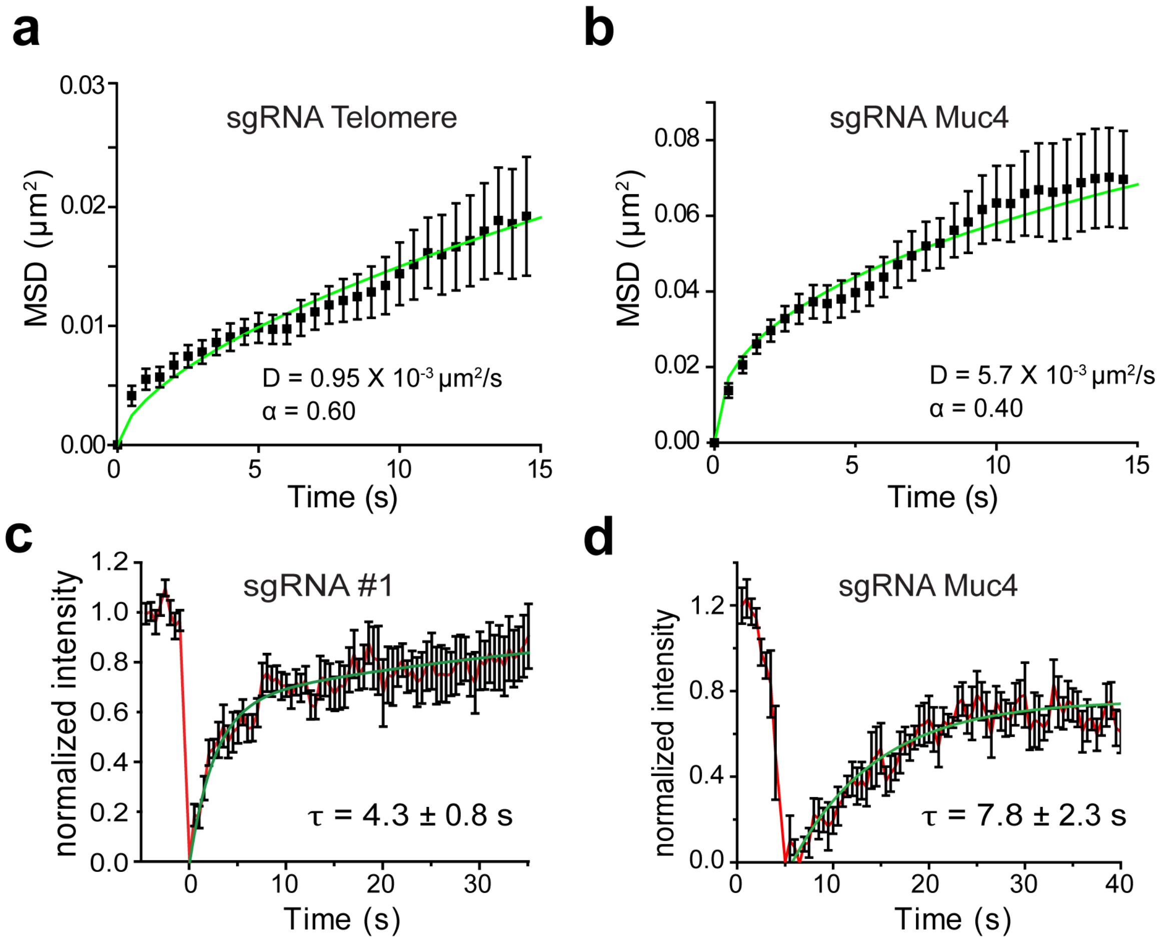

Supplementary Figure 5: FRAP and MSD analysis of dCas9 spots. **(a-b)** MSD plots of dCas9-GFP spots targeting telomeres (a) and the repetitive region in MUC4 (b) in HeLa cells. The green curve represents a fit to a two-dimensional random walk with a time exponent α (N_{cells} = 24). **(c-d)** FRAP analysis of dCas9-GFP targeting telomeres (N_{cells} = 14) (c) and MUC4 (N_{cells} = 36) (d) in stable dCas9-GFP U2OS cells. Data was fit to a single exponential function (green curve) to calculate the lifetime of fluorescence recovery. Error bars show s.e.m.

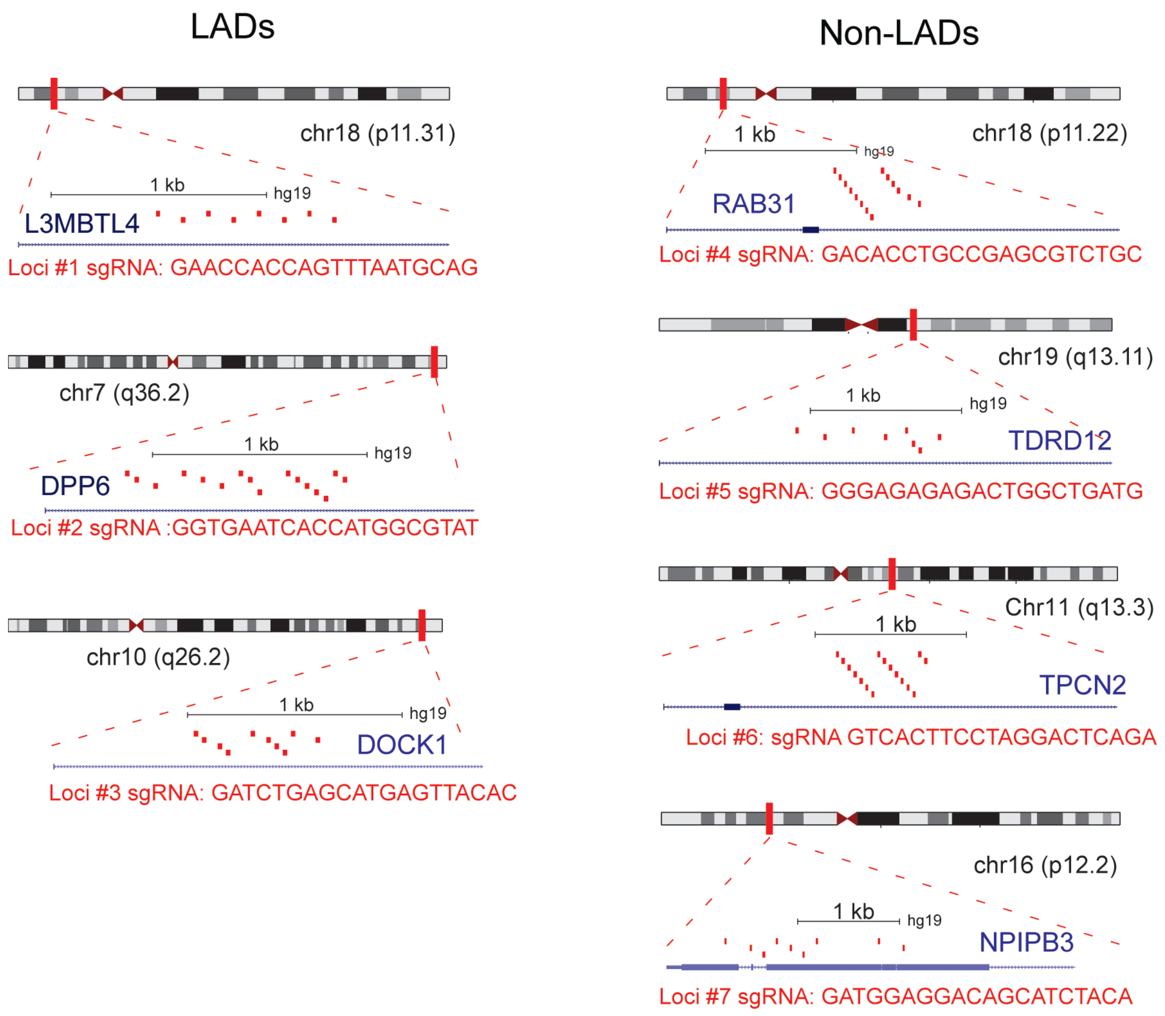

Supplementary Figure 6: Positions of LAD and non-LAD chromatin regions used for imaging. sgRNA targeted LAD and non-LADs and respective number of unique sgRNA targeted repeats are highlighted in red. Overlapping Refseq genes are shown in blue.

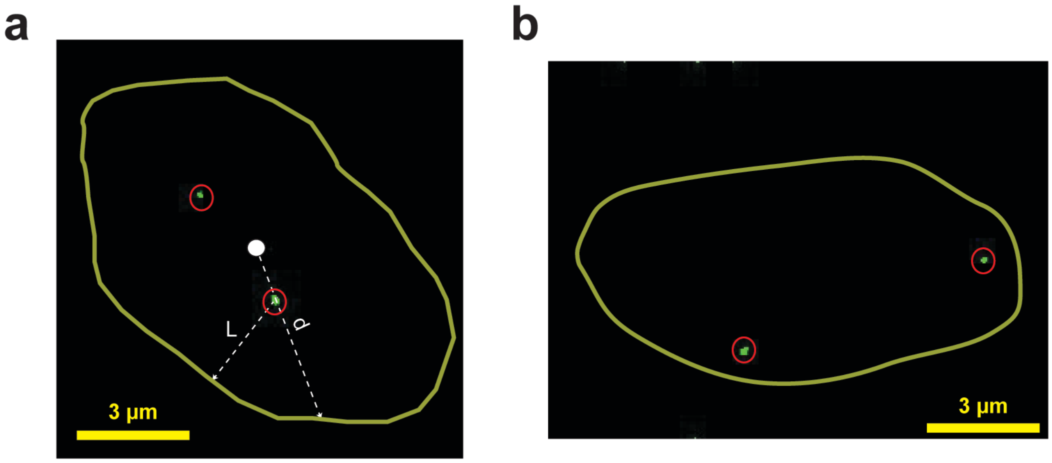

Supplementary Figure 7: Determining the relative positions of LAD and non-LAD chromatin regions to nuclear periphery. (a-b) Representative images of stable dCas9-GFP U2OS cells transduced with LAD #2 (b) and non-LAD #7 (c). The nuclear periphery was marked with yellow. The distance of each spot (red circles) to the nuclear periphery has been calculated as the shortest distance from the spot to the nuclear membrane (L). This distance was normalized to the length (d) of the line drawn from the center of the nucleus (white dot) to the nuclear periphery passing through the analyzed spot.

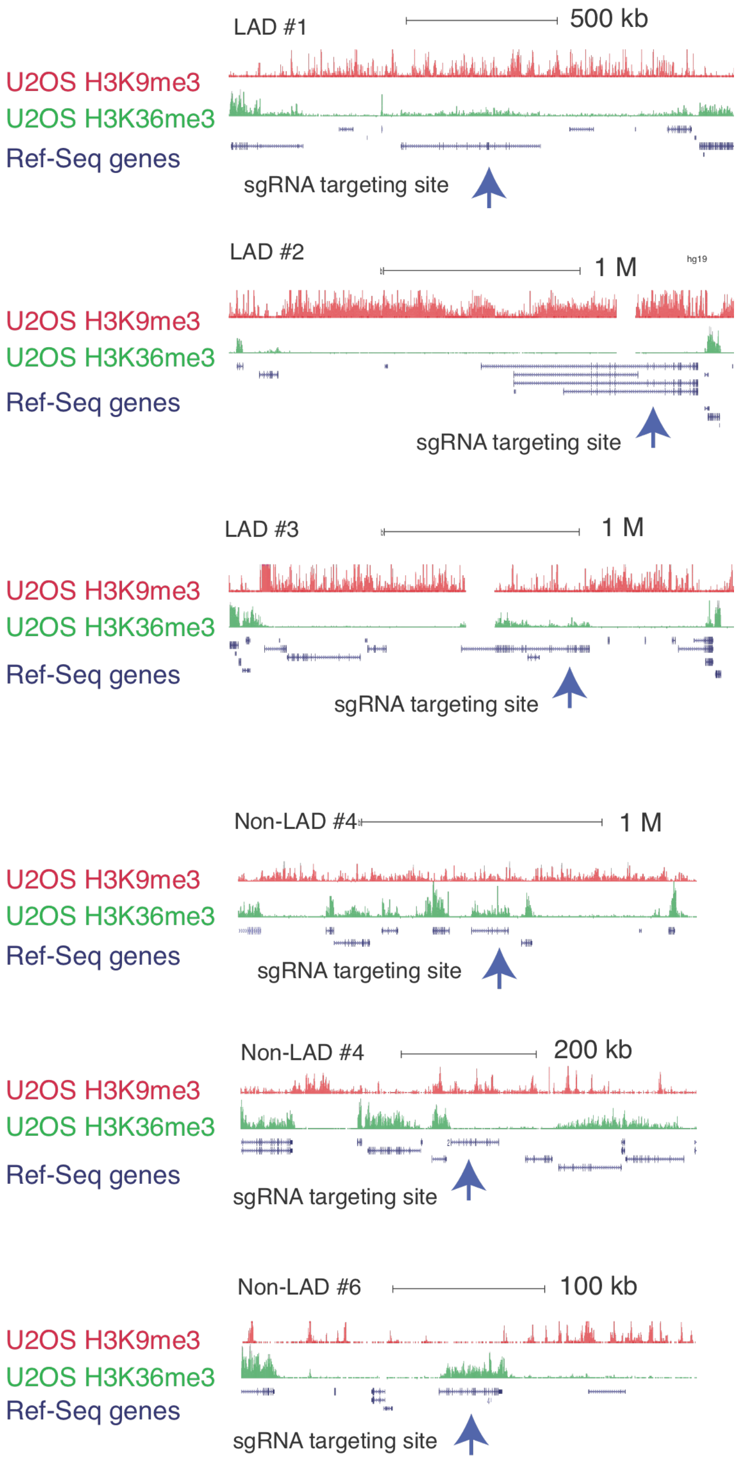

Supplementary Figure 8: The ENCODE chromatin state tracks show ChIP-Seq signal intensities for H3K9me3 (GEO: GSM788078) and H3K36me3 (GEO: GSM788076) histone modification marks in U20S cells. The Ref-Seq gene positions are shown in blue color under the track images.

3. **Supplementary Movie Legends**

Supplementary Movie 1. Transduction efficiency of lentivirus targeting centromere. Stable dCas9-GFP U2OS cells were transduced with an sgRNA lentivirus targeting centromeric satellite repeats. The cells were imaged using HiLo microscopy and moved to different fields of view manually. The scale bar is 9.6 µm.

Supplementary Movie 2. Transduction efficiency of lentivirus targeting MUC4 repetitive region. Stable dCas9-GFP U2OS cells were transduced with an sgRNA lentivirus targeting an 84-repeat sequence in the MUC4 gene. The cells were imaged using HiLo microscopy and moved to different fields of view manually. The scale bar is 9.6 µm.

Supplementary Movie 3. Lattice light sheet imaging of telomeres in a stable dCas9-GFP U2OS cell. U2OS cells stably expressing dCas9-GFP and MCP-mCherry were transduced with an sgRNA lentivirus targeting telomeres. The cells were imaged under lattice light sheet microscopy at 100 ms per frame. The scale bar is 6 µm.

Supplementary Movie 4. Lattice light sheet imaging of MUC4 non-repetitive region with 4 sgRNA 2.0 16x-MS2. U2OS cells stably expressing dCas9-GFP and MCP-mCherry were imaged using lattice light sheet microscopy at 100 ms per frame. The left panel shows a control stable cell without sgRNA transduction and the cell shown in the right panel was transduced with four unique sgRNA 2.0 16x-MS2 lentivirus targeting MUC4 non-repetitive region. The dCas9-GFP signal is not observable and only MCP-mCherry signal is shown. The scale bar is 6 µm.

Supplementary Movie 5. Long term imaging of dCas9-sgRNA complexes localized to locus #1 in a stable dCas9-GFP U2OS cell. Cells were transduced with sgRNA #1 lentivirus and imaged with HiLo microscopy at 50 ms per frame. The scale bar is 6 µm.

Supplementary Movie 6. Real time observation of replication of genomic loci in different chromosomes in HeLa cells. Cells were co-transfected with sgRNA 14x-MS2 #1. dCas9-mCherry, and MCP-YFP and imaged using scanning confocal microscopy at every 15 minutes. DNA replication of the same genomic locus in different chromosomes

was observed in different frames. See Figure 5a for the analysis of this movie. The scale bar is 3 μm.

Supplementary Movie 7. Single particle tracking of dCas9-mCherry localized to locus #1 in a HeLa cell. Cells were co-transfected with an sgRNA 14x-MS2 targeting locus #1, dCas9-mCherry and MCP-YFP, and imaged using scanning confocal microscopy at 100 ms per frame. Tracking of each spot to a 2D Gaussian is shown per frame and center of the Gaussian is highlighted with a colored circle. The scale bar is 6 μm.

Supplementary Movie 8. FRAP measurements of dCas9-GFP localized to telomeres with partial recovery in stable dCas9-GFP U2OS cells. Cells were transduced with sgRNA telomere lentivirus and imaged with HiLo microscopy at 300 ms per frame. Telomeres highlighted with colored ellipses were photobleached using a focused 488 nm beam. Telomeres marked with green ellipses did not show any detectable recovery over the course of the movie. The telomere marked with a red ellipse showed partial recovery. Scale bar is 6 μm.

Supplementary Movie 9. FRAP measurements of dCas9-GFP localized to telomeres without recovery in stable dCas9-GFP U2OS cells. Cells were transduced with sgRNA telomere lentivirus and imaged with HiLo microscopy at 300 ms per frame. Telomeres highlighted with a red ellipse were photobleached using a focused 488 nm beam. No recovery has been observed for these spots. Scale bar is 6 μm.
